# Emerging COVID-19 coronavirus: glycan shield and structure prediction of spike glycoprotein and its interaction with human CD26

**DOI:** 10.1080/22221751.2020.1739565

**Published:** 2020-03-17

**Authors:** Naveen Vankadari, Jacqueline A. Wilce

**Affiliations:** Monash Biomedicine Discovery Institute and Department of Biochemistry and Molecular Biology, Monash University, Clayton, Australia

**Keywords:** Coronavirus, CD26, glycosylation, DPP4, spike glycoprotein, docking

## Abstract

The recent outbreak of pneumonia-causing COVID-19 in China is an urgent global public health issue with an increase in mortality and morbidity. Here we report our modelled homo-trimer structure of COVID-19 spike glycoprotein in both closed (ligand-free) and open (ligand-bound) conformation, which is involved in host cell adhesion. We also predict the unique N- and O-linked glycosylation sites of spike glycoprotein that distinguish it from the SARS and underlines shielding and camouflage of COVID-19 from the host the defence system. Furthermore, our study also highlights the key finding that the S1 domain of COVID-19 spike glycoprotein potentially interacts with the human CD26, a key immunoregulatory factor for hijacking and virulence. These findings accentuate the unique features of COVID-19 and assist in the development of new therapeutics.

An outbreak of potentially lethal coronavirus (named COVID-19) in Wuhan, China, is spreading globally and impacting millions of people geographically linked with the Chinese population [1]. Current evidence suggests that the virus originated from wild animals and birds (https://www.cdc.gov/coronavirus/) [2]. To date, more than 2,800 deaths and 87,000 confirmed positive cases have been reported around the world, making COVID-19 a major health concern. As a first line of treatment, along with the antiviral drugs, clinicians are using SARS-CoV and MERS-CoV neutralizing antibodies targeting the S1 domain of the COVID-19 spike glycoprotein [1]. Very recently (25 January 2020) the first and complete genome sequence of COVID-19 was deposited in the NCBI (GenBank: MN908947.3) providing the key to the likely structure and glycosylation pattern of the viral proteins and consequent mode of interaction with the host cell. Similar to most other coronaviruses, the outer membrane spike glycoprotein, known for its glycosylation [3], is the prime host interacting protein with host cell targets (such as ACE2, CD26, Ezrin, cyclophilins and other cell adhesion factors) important for cell adhesion and virulence [4,5]. However, the specific host cell factors or proteins that facilitate the novel COVID-19 remain elusive. The current study was thus undertaken to predict the COVID-19 spike glycoprotein structure and glycan shield pattern that has great implications for understanding the viral camouflage and mode of cell entry, potentially assisting the development of new vaccines, antibodies, small-molecule drugs and screening of the human host targets.

The Clustal-W sequence alignment of COVID-19 and SARS-CoV spike glycoproteins (Figure S1) shows ∼91% identity in the S2 domain region (aa570–aa1278), however it lacks similarity in three regions (aa677–690, wing), (aa877–884 and aa930–943, stalk). A larger sequence difference (∼55% identity), was found in the S1 domain (aa01–aa550), which is known for its host cell target interaction underlying cell adhesion and virulence [4,5]. Despite sequence dissimilarity in the S1 domains there are conserved residues involved in ternary folding which were conserved. This suggests that the COVID-19 might interact with some of the previously known host targets (ACE2, CD26, Ezrin, cyclophilins), albeit via slightly varied molecular interactions. Recent studies also support the possibility of COVID-19 and ACE-2 interaction [6].

To better understand the structure of COVID-19, including the position and orientation of unique residues involved in target binding, we modelled the homo-trimer structure of COVID-19 S1 and S2 domains (spike glycoprotein) using SWISS-MODEL (https://swissmodel.expasy.org/) using the structure of SARS-CoV (PDB: 6ACD) [4]. This model was validated using the C-Score (confidence score) and TM score (structural similarity) (Figure S2) demonstrating the most correct fold and confidence of the predicted structure. Further validation and refinement was completed by ensuring that the residues occupied Ramachandran favoured positions using Coot (www.mrc-imb.cam.uk/) (Figure S2). All amino acid residues were positioned according to their lowest energy possible orientation in the final model. The final modelled homotrimer structure of COVID-19 in C3 symmetry (Figure 1(A)) superimposes with SARS-CoV with a 0.85Å Cα RMSD and with a number of unique residues exposed on the surface COVID-19. A second modelled structure of COVID-19 spike glycoprotein, in ligand-bound conformation (Figure 1(B)) was also predicted based on the SARS-CoV/ACE2 complex structure (PDB:6ACG) [4]. This shows S1 domains in an open conformation, enabling it to interact with target host proteins. As is the case for other coronaviruses [7], we also identified 3C-like proteinase cleavage site (TGRLQ^SLQTY) (aa 997–1007) in COVID-19 spike glycoprotein using a server (https://services.healthtech.dtu.dk/). This 3C-like proteinase cleavage site could represent a site for drug discovery as currently being proposed for SARS-CoV [7].

**Figure 1.**
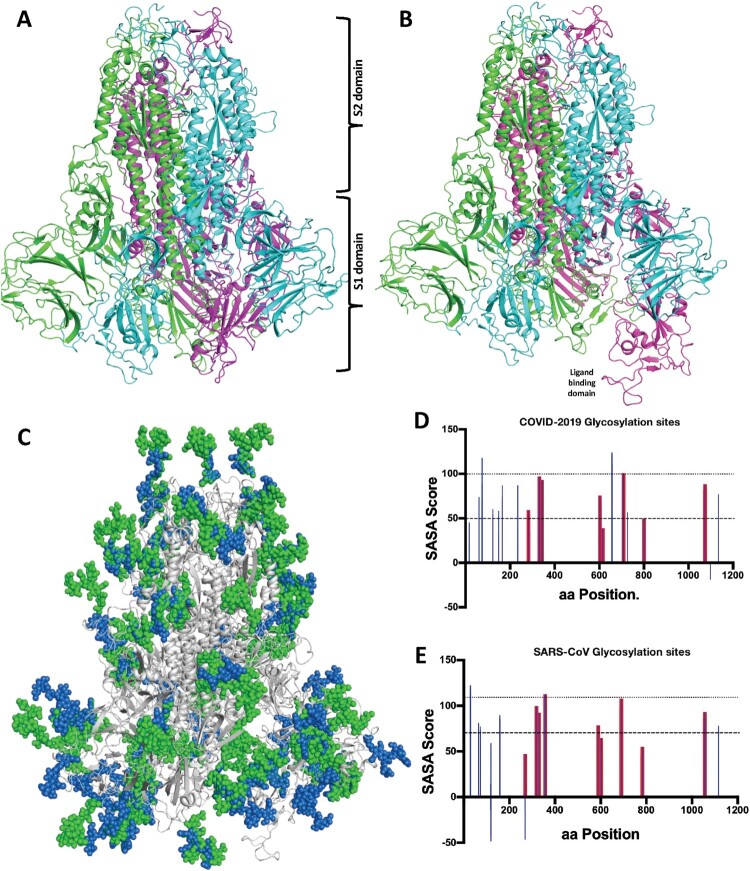
Overall homo-trimer model structure of the COVID-19 spike glycoprotein (A) ligand unbound conformation (B) ligand-bound conformation. The three protomers are coloured pink, green and cyan. S1- and S2- domains labelled. Receptor-binding induced hinge motion of S1 is distinguishable. (C) Predicted Glycan shield (spheres) of COVID-19 (green) and SARS-CoV (blue) spike glycoproteins. predicted 3C-like proteinase cleavage site (yellow). Predicted N-linked glycosylation sites for COVID-19 (D) and SARS-CoV (E). Unique glycosylation sites are coloured in *Blue*, and shared sites are shaded in *Red*.

To understand the glycosylation pattern and glycan shield of viral camouflage we used the (https://services.healthtech.dtu.dk/) and (http://glycam.org/) servers to predict N- and O-linked glycosylation sites on the surface of the modelled homo-trimer structure of COVID-19 spike glycoprotein and verified them according to their Solvent Accessible Surface Area (SASA) (Table S1). The spike glycoprotein trimer was then subjected to a surface glycosylation builder (http://glycam.org/glycoprotien_builder/) for the predicted sites and visualized in PyMol. We also performed the same analysis for the SARS-CoV spike protein, to identify significant differences in glycosylation patterns (Figure 1(D,E)). The built glycosylation shield structures of COVID-19 and SARS-CoV spike glycoproteins were superimposed and are shown in Figure 1(C). As shown in Figure 1(C) and Table S1, there are a number of conserved glycosylation sites between these two viral strains, however there are also several unique glycosylation sites in COVID-19 compared to SARS-CoV spike glycoprotein. This suggests a different shielding or glycan camouflage pattern of the spike proteins, which may underlie differences in host immunity. This leads to the intriguing question of whether COVID-19 could be responsive to a similar therapeutic approach to SARS [8].

Coronavirus trafficking into and hijacking the host cell is primarily driven by the N-terminal S1 domain of spike glycoprotein that interacts with several host cell proteins [4,5]. The host CD26 receptor cleaves amino-terminal dipeptides from polypeptides with either L-proline or L-alanine in the penultimate position, leading to T-cell activation and thus acting as a key immunoregulatory factor in viral infections [9]. Considering the current public health crisis, we considered the potential molecular interactions between COVID-19 spike protein and human CD26, with an interest to explore the structural differences or similarities between SARS-CoV and COVID-19 spike protein interactions. To this end, a computational model based selective docking was performed using the server Cluspro protein–protein docking (Www.cluspro.bu.edu) and Frodock (http://frodock.chaconlab.org/) for further validation using our modelled 3D homotrimer structure of COVID-19 Spike glycoprotein (Figure 2) and the human CD26 (PDB: 4QZV) [10]. The binding free energies were taken into consideration for selecting the best possible model. The final rigid docked complex structure was compared with the initial full-length COVID-19 spike glycoprotein and CD26 and their overall RMSD’s were found to be 1.34 and 0.28 Å for Cα atoms, respectively.

**Figure 2.**
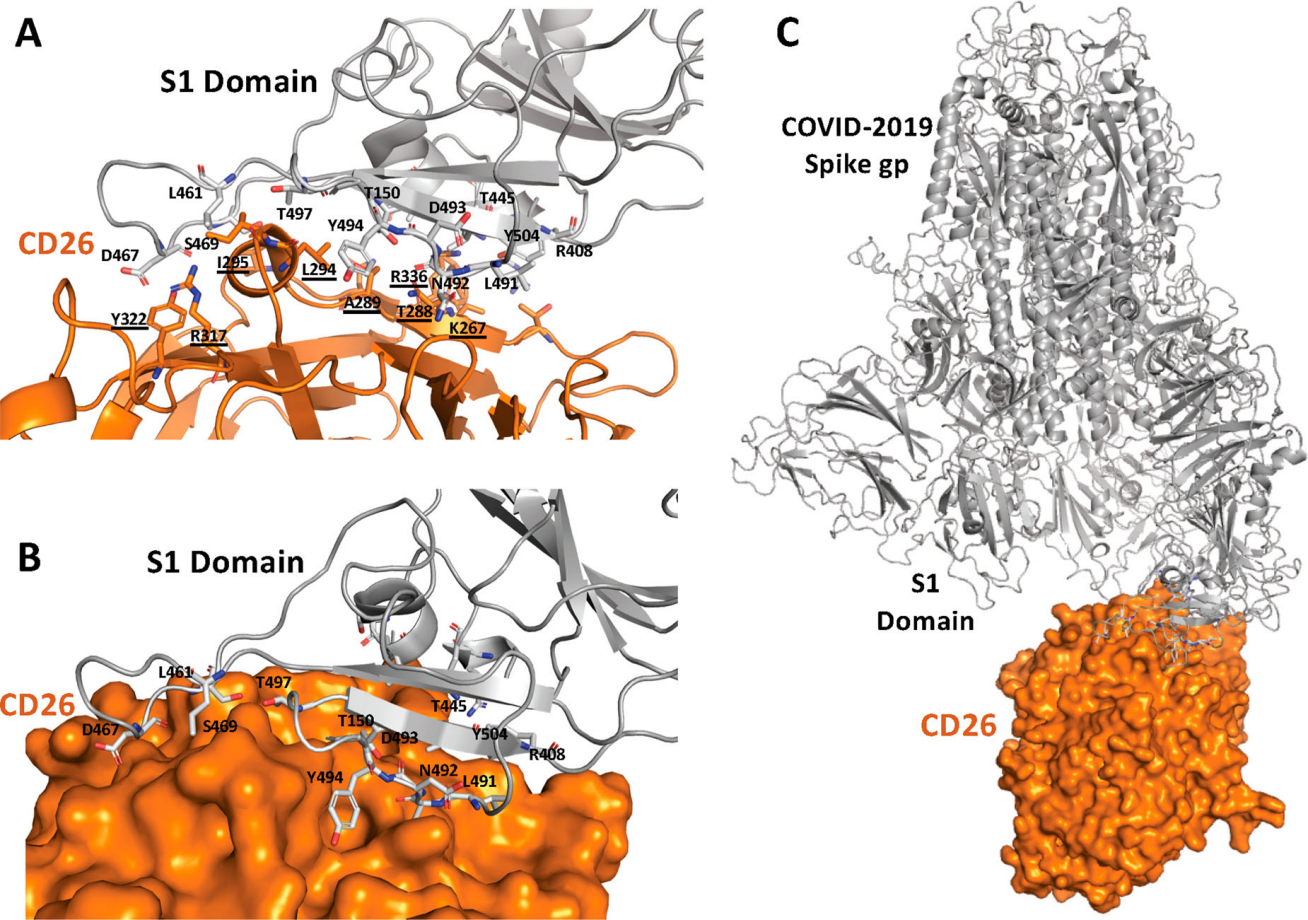
(A and B) Ribbon and a surface diagram showing the docking interface of modelled COVID-19 (grey) and human CD26 (orange)(PDB: 4QZV) complex. Predicted key residues involved in the interaction are shown in sticks (CD26 residues are underlined) (C) Overall docking results showing the surface model of CD26 with COVID-19 predicted homo-trimer structure (ligand-bound conformation).

The docked complex model of COVID-19 spike glycoprotein and CD26 (Figure 2) shows a large interface between the proteins. This suggests a possible tight interaction between the S1 domain loops in the modelled structure and the CD26 surface. Previous studies of CD26 binding have shown that residues K267, T288, A289, A291, L294, I295, R317, Y322 and D542 interact with Bat-CoV (MERS) spike protein [10]. Interestingly our docked model supports this despite the variability between these spike proteins’ S1 domains, with the same CD26 residues in close proximity to the active region of S1 domain in COVID-19. We also observed additional residues (Q286, I287, N338, V341, R336) of CD26 predicted to interact with the S1 domain of the spike protein via van der Waals or by hydrogen bonding. However, regarding the COVID-19 spike glycoprotein, we noticed many different and unique residues (R408, Q409, T445, V417, L461, D467, S469, L491, N492, D493, Y 494, T497, T150, Y504) predicted to interact with CD26. Some of these unique residues of S1 domain are also predicted interact with the ACE2 protein [6]. This underlines the novelty and uniqueness of COVID-19 and its interaction with human target proteins. This observation guides us to suggest that COVID-19 may share infection modes with that of SARS-CoV and MERS-CoV and that interactions with other targets also warrant investigation.

## Supplementary Material

Supplemental Material
