# Supplementary material for: Emerging COVID-19 coronavirus: glycan shield and structure prediction of spike glycoprotein and its interaction with human CD26
Source: Emerg Microbes Infect. 2020 Mar 17;9(1):601–4. doi: 10.1080/22221751.2020.1739565 (PMC7103712; doi:10.1080/22221751.2020.1739565)
Supplement: Supplemental Material [file TEMI_A_1739565_SM1730.zip › Table_S1_final.docx]

|  | |  | |  | |  | |  |  | |  | |  | |  | | | |  | |
| --- | --- | --- | --- | --- | --- | --- | --- | --- | --- | --- | --- | --- | --- | --- | --- | --- | --- | --- | --- | --- |
| **N-linked Glycan of COVID-19 and SARS-CoV** | | | | | | | | | | | | | | | | | |  | |  |
|  | |  | |  | |  | |  |  | |  | |  | |  | | | |  | |
| **COVID-2019** | | | | | | | |  | **SARS-CoV** | | | | | | | | | |  | |
| **Sequence** | **aa Chain** | | **aa No.** | | **SASA** | |  | **Sequence** | | **aa Chain** | | **aa No.** | | **SASA** | |  |  |  |  |  |
| CVN*LT | B | | 17 | | 45.2 + | |  | APN*YT | | A | | 29 | | 122.6 + | |  |  |  |  |  |
| CVN*LT | A | | 17 | | 44.7 + | |  | APN*YT | | B | | 29 | | 120.6 + | |  |  |  |  |  |
| CVN*LT | C | | 17 | | 23.2 | |  | APN*YT | | C | | 29 | | 114.9 + | |  |  |  |  |  |
| FSN*VT | C | | 61 | | 73.7 + | |  | YSN*VT | | C | | 65 | | 81.2 + | |  |  |  |  |  |
| FSN*VT | B | | 61 | | 73.2 + | |  | YSN*VT | | B | | 65 | | 80.8 + | |  |  |  |  |  |
| FSN*VT | A | | 61 | | 72.6 + | |  | YSN*VT | | A | | 65 | | 79.2 + | |  |  |  |  |  |
| GTN*GT | A | | 74 | | 70.5 + | |  | TIN*HT | | C | | 73 | | 77.3 + | |  |  |  |  |  |
| GTN*GT | C | | 74 | | 117.9 + | |  | TIN*HT | | A | | 73 | | 73.2 + | |  |  |  |  |  |
| GTN*GT | B | | 74 | | 87.8 + | |  | TIN*HT | | B | | 73 | | 70.5 + | |  |  |  |  |  |
| VNN*AT | A | | 122 | | 54.9 + | |  | IIN*NS | | A | | 118 | | 14.8 - | |  |  |  |  |  |
| VNN*AT | C | | 122 | | 60.5 + | |  | IIN*NS | | B | | 118 | | 13.7 - | |  |  |  |  |  |
| VNN*AT | B | | 122 | | 47.2 + | |  | INN*ST | | A | | 119 | | 64.4 + | |  |  |  |  |  |
| KNN*KS | B | | 149 | | 32.2 | |  | INN*ST | | C | | 119 | | 62.4 + | |  |  |  |  |  |
| KNN*KS | C | | 149 | | 53.7 + | |  | INN*ST | | B | | 119 | | 59.0 + | |  |  |  |  |  |
| KNN*KS | A | | 149 | | 58.3 + | |  | AFN*CT | | B | | 158 | | 89.7 + | |  |  |  |  |  |
| ANN*CT | A | | 165 | | 74.7 + | |  | AFN*CT | | A | | 158 | | 85.6 + | |  |  |  |  |  |
| ANN*CT | B | | 165 | | 86.7 + | |  | AFN*CT | | C | | 158 | | 83.9 + | |  |  |  |  |  |
| ANN*CT | C | | 165 | | 72.2 + | |  | DEN*GT | | A | | 269 | | 47.3 + | |  |  |  |  |  |
| GIN*IT | C | | 234 | | 18.2 - | |  | DEN*GT | | B | | 269 | | 46.5 + | |  |  |  |  |  |
| GIN*IT | B | | 234 | | 87.2 + | |  | DEN*GT | | C | | 269 | | 16.8 - | |  |  |  |  |  |
| GIN*IT | A | | 234 | | 38.7 | |  | FPN*IT | | B | | 318 | | 99.9 + | |  |  |  |  |  |
| NEN*GT | C | | 282 | | 59.2 + | |  | FPN*IT | | A | | 318 | | 91.9 + | |  |  |  |  |  |
| NEN*GT | A | | 282 | | 53.7 + | |  | VFN*AT | | B | | 330 | | 92.4 + | |  |  |  |  |  |
| NEN*GT | B | | 282 | | 35 | |  | VFN*AT | | A | | 330 | | 81.6 + | |  |  |  |  |  |
| FPN*IT | A | | 331 | | 96.9 + | |  | VFN*AT | | C | | 330 | | 78.4 + | |  |  |  |  |  |
| FPN*IT | C | | 331 | | 65.5 + | |  | LYN*ST | | C | | 357 | | 112.7 + | |  |  |  |  |  |
| FPN*IT | B | | 331 | | 96.2 + | |  | LYN*ST | | B | | 357 | | 110.8 + | |  |  |  |  |  |
| VFN*AT | B | | 343 | | 93.0 + | |  | LYN*ST | | A | | 357 | | 104.9 + | |  |  |  |  |  |
| VFN*AT | C | | 343 | | 92.5 + | |  | GTN*AS | | B | | 589 | | 78.6 + | |  |  |  |  |  |
| VFN*AT | A | | 343 | | 83.9 + | |  | GTN*AS | | A | | 589 | | 78.1 + | |  |  |  |  |  |
| GTN*TS | A | | 603 | | 75.5 + | |  | GTN*AS | | C | | 589 | | 66.3 + | |  |  |  |  |  |
| GTN*TS | B | | 603 | | 72.3 + | |  | DVN*CT | | B | | 602 | | 64.6 + | |  |  |  |  |  |
| GTN*TS | C | | 603 | | 63.9 + | |  | DVN*CT | | A | | 602 | | 60.5 + | |  |  |  |  |  |
| DVN*CT | C | | 616 | | 38.6 | |  | DVN*CT | | C | | 602 | | 53.0 + | |  |  |  |  |  |
| DVN*CT | B | | 616 | | 38.6 | |  | YSN*NT | | B | | 691 | | 107.8 + | |  |  |  |  |  |
| DVN*CT | A | | 616 | | 20.4 | |  | YSN*NT | | C | | 691 | | 100.1 + | |  |  |  |  |  |
| HVN*NS | B | | 657 | | 123.9 + | |  | YSN*NT | | A | | 691 | | 97.2 + | |  |  |  |  |  |
| HVN*NS | C | | 657 | | 121.7 + | |  | GFN*FS | | A | | 783 | | 55.3 + | |  |  |  |  |  |
| HVN*NS | A | | 657 | | 111.9 + | |  | GFN*FS | | B | | 783 | | 52.6 + | |  |  |  |  |  |
| YSN*NS | A | | 709 | | 95.2 + | |  | GFN*FS | | C | | 783 | | 45.5 + | |  |  |  |  |  |
| YSN*NS | B | | 709 | | 100.6 + | |  | ERN*FT | | A | | 1056 | | 93.1 + | |  |  |  |  |  |
| YSN*NS | C | | 709 | | 95.8 + | |  | ERN*FT | | B | | 1056 | | 86.7 + | |  |  |  |  |  |
| PTN*FT | A | | 717 | | 51.0 + | |  | ERN*FT | | C | | 1056 | | 84.8 + | |  |  |  |  |  |
| PTN*FT | B | | 717 | | 56.6 + | |  | IIN*NT | | B | | 1116 | | 78.3 + | |  |  |  |  |  |
| PTN*FT | C | | 717 | | 40.9 + | |  | IIN*NT | | A | | 1116 | | 76.7 + | |  |  |  |  |  |
| GFN*FS | C | | 801 | | 49.7 + | |  | IIN*NT | | C | | 1116 | | 76.4 + | |  |  |  |  |  |
| GFN*FS | A | | 801 | | 48.2 + | |  |  | |  | |  | |  | |  |  |  |  |  |
| GFN*FS | B | | 801 | | 48.2 + | |  |  | |  | |  | |  | |  |  |  |  |  |
| EKN*FT | B | | 1074 | | 83.1 + | |  |  | |  | |  | |  | |  |  |  |  |  |
| EKN*FT | C | | 1074 | | 81.2 + | |  |  | |  | |  | |  | |  |  |  |  |  |
| EKN*FT | A | | 1074 | | 88.2 + | |  |  | |  | |  | |  | |  |  |  |  |  |
| VSN*GT | B | | 1098 | | 18.9 - | |  |  | |  | |  | |  | |  |  |  |  |  |
| VSN*GT | A | | 1098 | | 17.9 - | |  |  | |  | |  | |  | |  |  |  |  |  |
| VSN*GT | C | | 1098 | | 14.6 - | |  |  | |  | |  | |  | |  |  |  |  |  |
| IVN*NT | C | | 1134 | | 73.2 + | |  |  | |  | |  | |  | |  |  |  |  |  |
| IVN*NT | A | | 1134 | | 77.0 + | |  |  | |  | |  | |  | |  |  |  |  |  |
| IVN*NT | B | | 1134 | | 74.6 + | |  |  | |  | |  | |  | |  |  |  |  |  |
|  |  | |  | |  | |  |  | |  | |  | |  | |  |  |  |  |  |
|  |  | |  | |  | |  |  | |  | |  | |  | |  |  |  |  |  |
| **O-linked Glycan of COVID-19 and SARS-CoV** | | | | | | | | | | | | | | | | | |  | |  |
|  | |  | |  | |  | |  |  | |  | |  | |  | | | |  | |
| **COVID-2019** | | | | | | | |  | **SARS-CoV** | | | | | | | | | |  | |
| **Sequence** | | **aa Chain** | | **aa No.** | | **SASA** | |  | **Sequence** | | **aa Chain** | | **aa No.** | | **SASA** | | | |  | |
| QPT*ES | | B | | 323 | | 43.4 + | |  | RCT*TF | | B | | 20 | | 30.6 | | | |  | |
| QPT*ES | | C | | 323 | | 42.1 + | |  | RCT*TF | | C | | 20 | | 23.8 | | | |  | |
| QPT*ES | | A | | 323 | | 29.1 | |  | RCT*TF | | A | | 20 | | 18.6 - | | | |  | |
| SPT*KL | | C | | 385 | | 86.1 + | |  | CTT*FD | | B | | 21 | | 47.6 + | | | |  | |
| SPT*KL | | A | | 385 | | 79.6 + | |  | CTT*FD | | A | | 21 | | 51.0 + | | | |  | |
| SPT*KL | | B | | 385 | | 73.1 + | |  | CTT*FD | | C | | 21 | | 44.0 + | | | |  | |
| NCT*EV | | B | | 618 | | 94.1 + | |  | WGT*SA | | C | | 247 | | 12.0 - | | | |  | |
| NCT*EV | | A | | 618 | | 78.2 + | |  |  | |  | |  | |  | | | |  | |
| NCT*EV | | C | | 618 | | 59.0 + | |  |  | |  | |  | |  | | | |  | |
| SMT*KT | | B | | 732 | | 26.3 | |  |  | |  | |  | |  | | | |  | |
| SMT*KT | | C | | 732 | | 26.3 | |  |  | |  | |  | |  | | | |  | |
| SMT*KT | | A | | 732 | | 25.5 | |  |  | |  | |  | |  | | | |  | |
|  | |  | |  | |  | |  |  | |  | |  | |  | | | |  | |


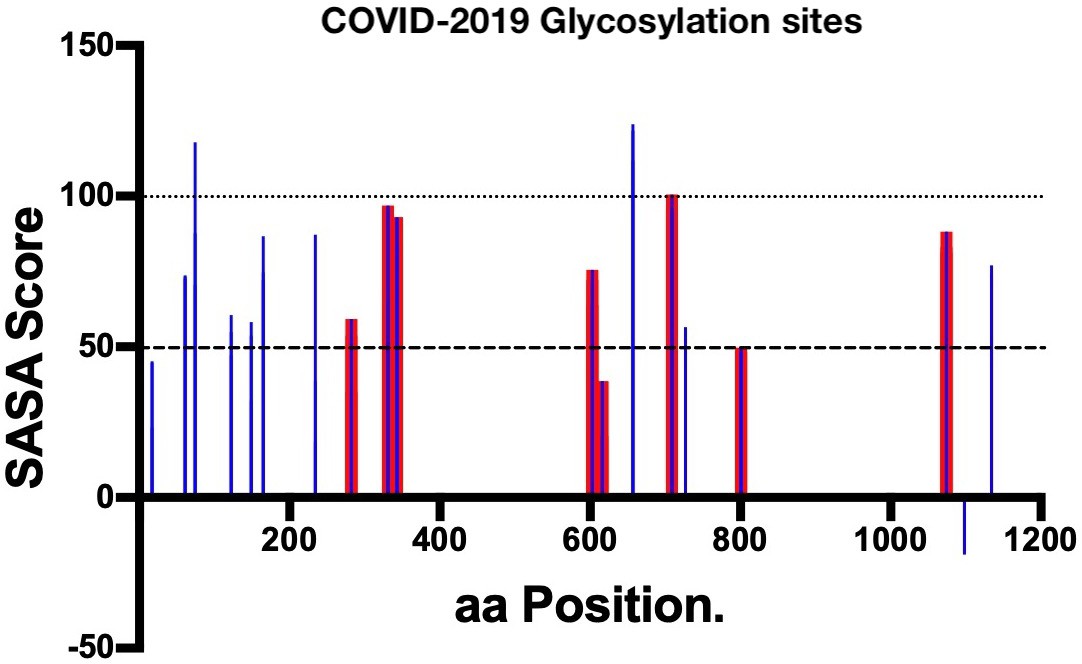

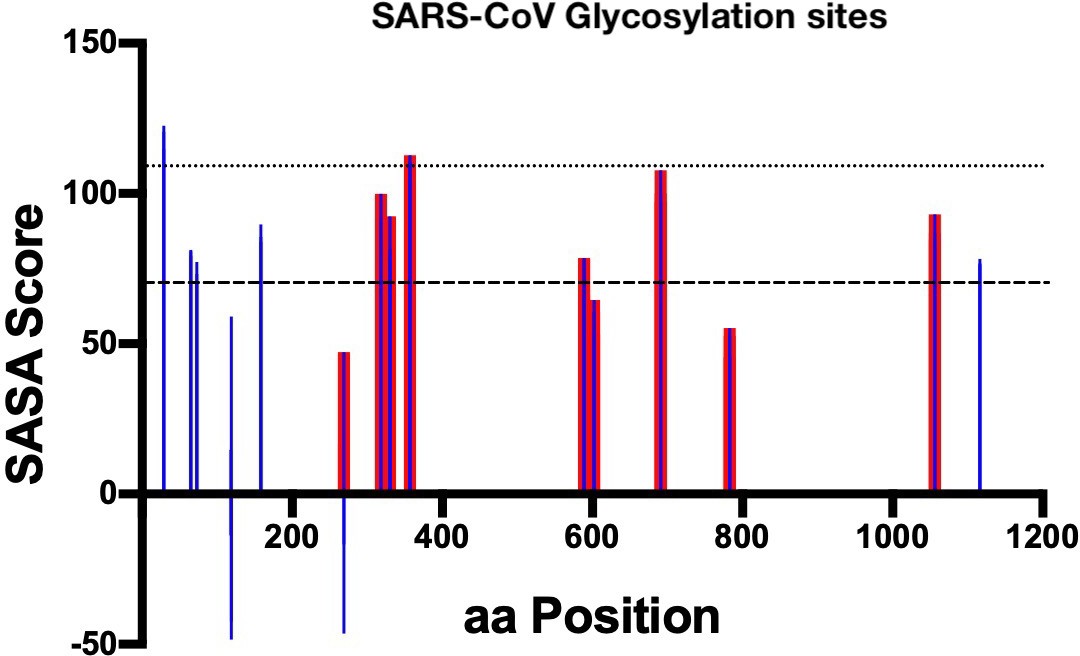


**A**

**B**

**C**

**D**
