# Supplementary figures and images for: Emerging COVID-19 coronavirus: glycan shield and structure prediction of spike glycoprotein and its interaction with human CD26
Source: Emerg Microbes Infect. 2020 Mar 17;9(1):601–4. doi: 10.1080/22221751.2020.1739565 (PMC7103712; doi:10.1080/22221751.2020.1739565)

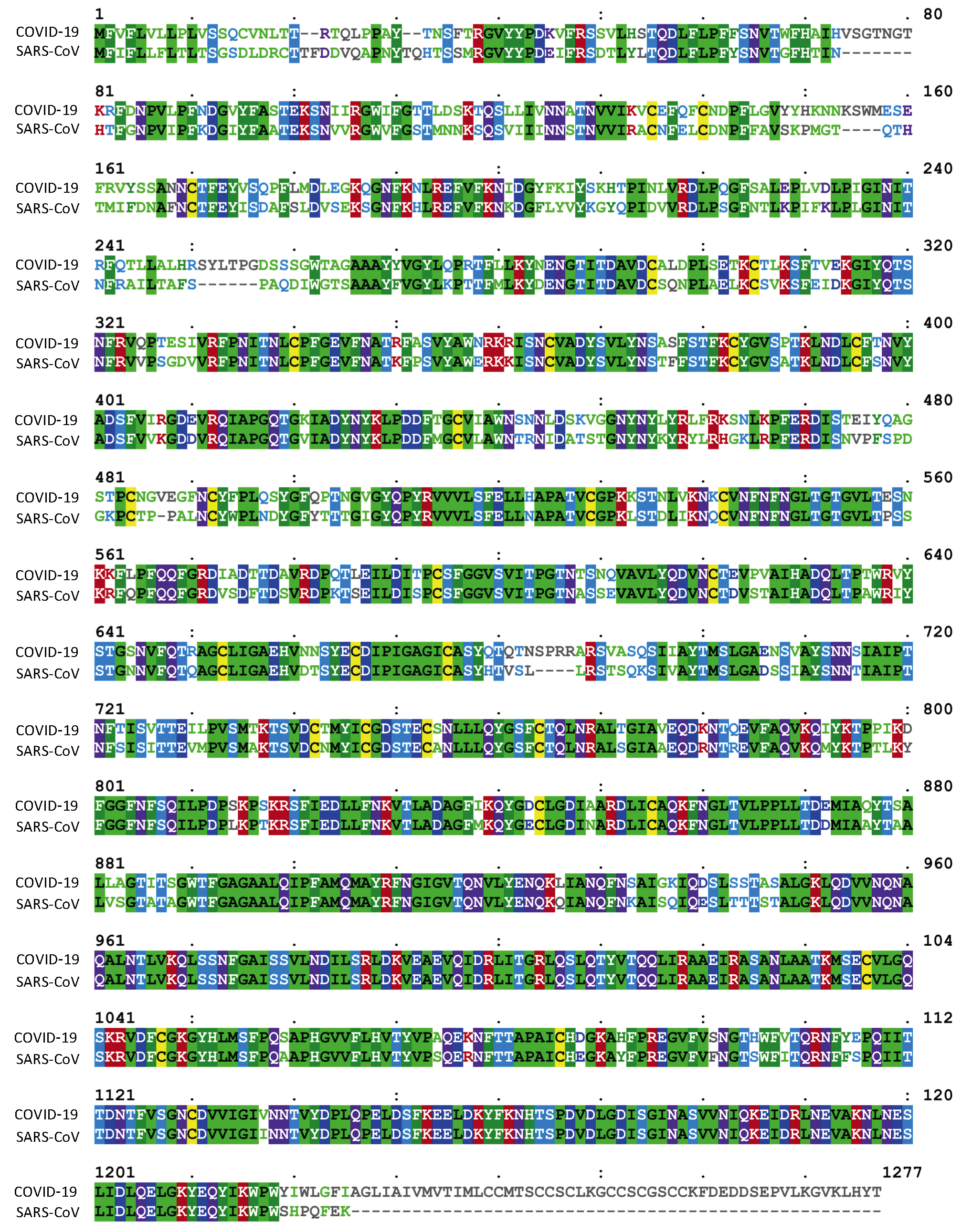

Supplement: Supplemental Material [file TEMI_A_1739565_SM1730.zip › Figure_S1_final.tiff]

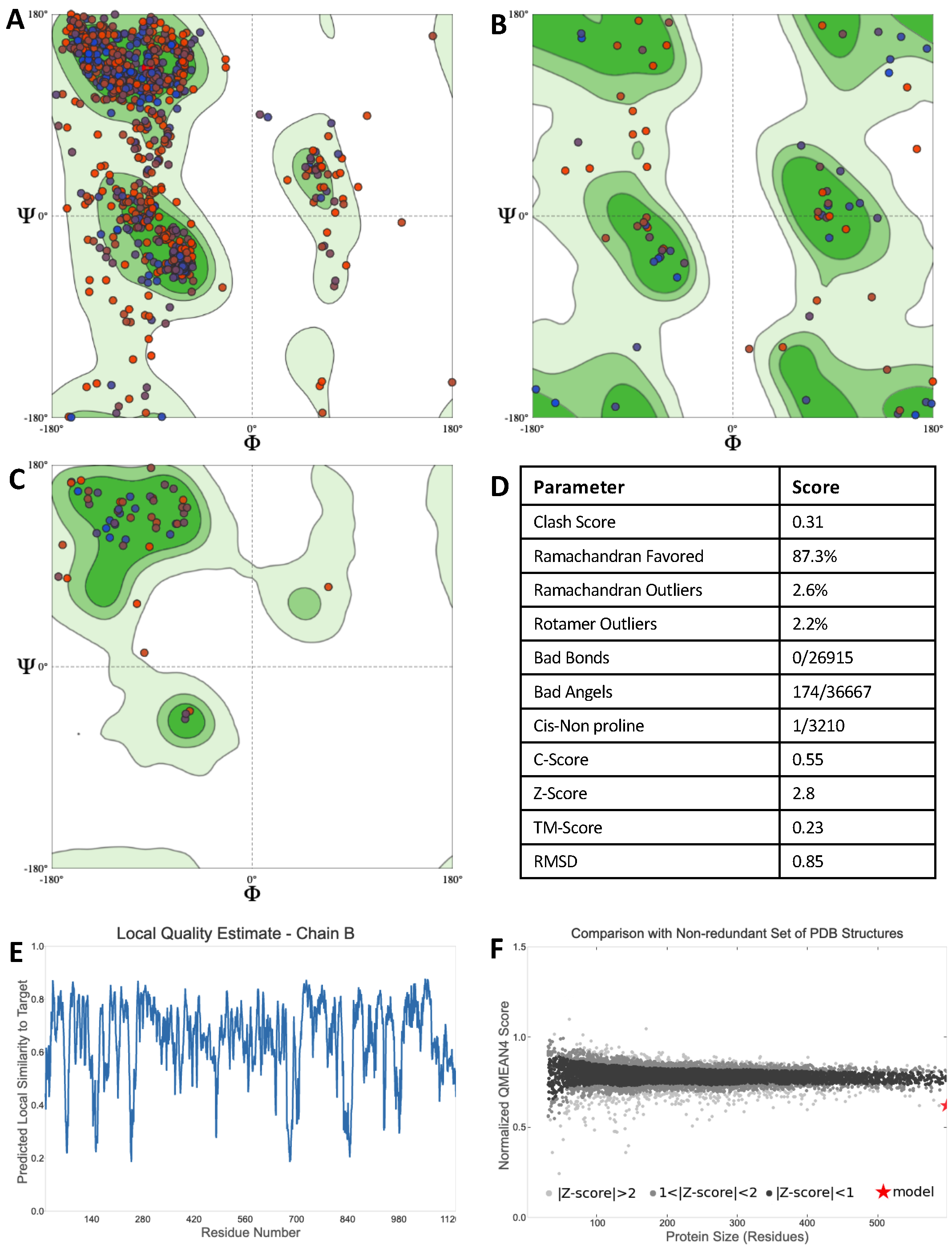

Supplement: Supplemental Material [file TEMI_A_1739565_SM1730.zip › Figure_S2_final.tiff]
